# Cases Presented to the Clinical Class in the Medical Wards of the Mercy Hospital, Feb. 11-12, 1875

**Published:** 1875-03-01

**Authors:** N. S. Davis

**Affiliations:** Professor of Clinical Medicine, etc.


### CASES PRESENTED TO THE CLINICAL CLASS IN THE MEDICAL WARDS OF THE MERCY HOSPITAL, FEBRUARY 11 AND 12, 1875.

BY N. S. DAVIS, M.D., PROFESSOR OF CLINICAL MEDICINE, ETC.

CASE I.—PLEURO-PNEUMONIA.—M. B——, aged thirty-five years, was admitted into the hospital February 8th, three days since, presenting an anxious expression of countenance; rather purplish flush on his cheeks; dryness of the lips; white coat on the tongue; pulse 110 per minute, moderately firm under the finger; respirations 40 per minute, short and quick; skin dry and temperature 102° F. He had a frequent, short, rather stifled cough, with scanty expectoration of tenacious mucus slightly tinged with blood. He complained of constant pain in the lower part of the right side of his chest, rendered very sharp and severe by coughing or attempting to take a full breath; his urine was scanty, bowels quiet, but no appetite and little thirst. Percussion indicated moderate dullness on the right side, extending from two to three inches above the diaphragm, and over the same space auscultation afforded slight pleuritic friction, and sub-crepitant rale, with diminished expansion of that side by inspiration. The patient had been sick only two days before admission into the hospital. After the members of the class had sufficiently examined the patient, their attention was directed to the essential items of diagnosis and the indications for treatment.

The pain in the lower part of the right side of the chest, rendered severe and sharp by coughing; the character of the expectoration; and the physical signs elicited by percussion and auscultation, all point distinctly to the existence of pneumonia in the lower lobe of the right lung, and pleuritic inflammation in the corresponding part of the pleura.

The blood in the sputa; the subcrepitant instead of the fine crepitant rale; and the imperfect inflation of that lung by inspiration, with decided dullness on percussion, indicated pneumonic exudation, constituting the second stage of the inflammatory process. During the three days that have elapsed since the patient came under observation, the dullness on percussion has extended higher, especially over the posterior part of that side, and the friction which was at first distinct has ceased to be audible; circumstances that indicate a moderate serous effusion from the inflamed surface of the pleura. We have as the present pathological conditions, a pleuro-pneumonia in the second stage of progress, in a patient whose blood is below the normal plasticity, and whose vital resistance to disease is impaired. The two last-mentioned conditions are shown by the comparative low temperature and dingy color of the surface; the quick and weak pulse; a frequency of respiration and sense of oppression in the chest out of proportion to the extent of the local inflammation; and the appearance of a large vesicle of pemphigus on the back of one hand. This apparent impairment of constitutional vigor may be owing in part to the effects of a severe attack of pneumonia which he suffered while in Europe a few years since, and in part to the effects of exposure to the unusually protracted and severe cold of our present winter.

*Treatment.*—Immediately after the patient came into the hospital a sinapism was applied to the right side long enough to induce some redness of the surface, and then the whole right half of the chest covered with emollient poultices which are still continued. He was also directed to take one teaspoonful of the following mixture every three hours:

| | |
|---|---|
| ℞—Ammo. Hydrochlo. | ʒ iii. |
| Ant. et. Pot. Tart. | 2 grs. |
| Morph. Sulph. | 3 grs. |
| Glycyrrhizæ Syrupi. | ℥ iv.—Mix. |

The next day (yesterday) he was suffering less pain in his side, but his breathing was more short and labored; lips and face more purple; pulse quicker and weaker; and the area of dullness on percussion over the lateral part of the right side of the chest increased. The doses of the anodyne expectorant mixture were directed to be continued only every four hours, and a powder containing two grains of sulphate of quinia, one of calomel, and one-sixth of a grain of sulphate of morphia, between. It was thought the quinine would check the further exudation by imparting more tone or contraction to the pulmonary vessels, and by exerting a general tonic influence. This treatment has been continued until to-day (Feb. 11th), accompanied by a steady improvement in the condition of the patient; and yet the present examination shows indications of much general weakness, dullness, and sub-mucous rhonchus over the affected side, and some blood in the expectoration. The abdomen is moderately distended with tympanitis, and, as there has been no intestinal evacuation during the last three days, he was directed to have castor oil, half an ounce, followed, if necessary, in three hours by an enema sufficient to move the bowels. The powders of quinine, two grains, and morphia, one-sixth of a grain, without the calomel, were continued every four hours, but the hydrochlorate of ammonia mixture was discontinued, and the following formula substituted therefor:

℞—Liquor Ammo. Acetatis, ℥ ii.
Syrup Ipecac ℥ ss.
Tinct. Opii et Camph. ℥ iss.
Carb. Ammo. ʒ iss.

Mix. Dose, one teaspoonful, or fluid drachm every four hours, alternating with the powders.

The patient was to have at short and regular intervals milk and meat broths for nourishment, and continuance of the emollient applications over the chest.*

CASE II. HÆMOPTYSIS FOLLOWED BY CASEOUS DEGENERATION.

Mr. C.—— is a laborer, aged about 28 years, medium size, and naturally strong and robust. He has been in the hospital about eight weeks, and has been two or three times examined by the clinical class.

The history of his case is briefly as follows: About fifteen months since, while in apparent good health, and engaged in active physical labor, he was taken suddenly with pretty active hæmorrhage from the lungs. This soon ceased, but was followed by some soreness in the infraclavicular region of the left side, and a moderate cough. He suffered, however, but little loss of flesh or strength until about five months later, when he began to suffer more pain and sense of oppression in the upper part of the chest, more severe cough, and some fever.

These symptoms he attributed to a severe cold. In a few days he began to expectorate suddenly a considerable quantity of thick yellow matter, mixed with some curdy substance, and an occasional mouthful of blood. The cough and purulent expectoration continued several weeks, accompanied by some emaciation and loss of strength, but gradually diminished until they had nearly ceased, when slight hæmorrhage was renewed. From that time he again improved until about two months since, when he was again attacked with pain and soreness in his chest, slight hæmorrhages, and a harsh, severe cough. Soon after the commencement of this attack, and while he was yet spitting up blood, he was admitted into the Mercy Hospital, and came directly under the observation of the clinical class. At that time, though a little thin in flesh, he had none of that pale, cachectic look common to patients suffering from tubercular phthisis, especially after repeated hæmorrhages. His pulse, respiration, temperature, and hue of the skin were nearly natural, and the chest retained its natural shape. Auscultation revealed simply a harsh or exaggerated respiratory murmur over the anterior part of the right side, with entire silence over the left side from the clavicle to the nipple, while over the remainder of the left and the whole of the posterior parts of the chest the respiratory murmur was natural, with now and then a coarse mucous rhonchus. Percussion yielded a clear, pulmonary resonance over all the chest except directly below the left clavicle, where it was entirely dull. The dullness, however, did not exist over half of the space from which the respiratory murmur was excluded, leaving the space from the lower margin of the second rib to the nipple, presenting the rare coincidence of clear resonance on percussion with the absence

*Feb. 17th, on visiting the ward, this patient was found convalescing. The only change in his treatment had been longer intervals between his medicines and more nourishment.

of all sounds in auscultation. Such a coincidence might result from either emphysema of a portion of the lung, or from temporary occlusion of a bronchus. After a careful comparison of the existing symptoms and physical signs with the past history of the case, it was thought that the original hæmorrhage, fifteen months since, resulted from the rupture of a vessel in the apex of the left lung, without any previous tubercular deposits; that extravasation of blood took place at the time into the tissue surrounding the point of rupture, which, during the succeeding five months, underwent first caseous and second purulent degeneration, the latter accompanied by cough, soreness, and finally the copious puruloid expectoration already described. From this circumscribed cavity, thus formed in the apex of that lung, all the subsequent hæmorrhages, and most of the expectoration, have come.

The hæmorrhage which was existing at the time of his admission had again filled this cavity with a fibrinous clot, temporarily preventing the entrance of air into the cavity or the adjacent portion of the lung, causing dullness over the former, and silence without dullness over the latter. He was directed to take a teaspoonful of two parts of fluid extract of hammamelis virginicus and one part of fluid extract of ergot every two hours until the hæmorrhage should cease, and a dose of some anodyne expectorant night and morning to lessen soreness and cough. In twenty-four hours the hæmorrhage ceased, and the ergot and witch-hazel were given at much longer intervals. In a few days the patient began to expectorate copiously a thick, yellow matter, and to complain of weakness and some sweating at night. At the same time it was found that the respiratory murmur had returned in the upper part of the left side of the chest, except directly below the clavicle, where there was some coarse rhonchus. The ergot mixture was then omitted, and a mixture of extract or syrup of malt and compound syrup of hypophosphites given instead.

For three weeks the patient continued to cough and expectorate freely, considerable quantities of purulent matter, and complained of shortness of breath and soreness at the upper part of the left side. Since then all his symptoms have steadily improved until now, February 12th, he has no yellow or purulent expectoration, only slight cough, and is gaining in flesh and strength. Yet there is dullness below the left clavicle, with a well-marked cavernous sound when the patient takes a full or forced inspiration. He may continue to improve until the injured part of the lung is entirely cicatrized, or he may have a return of hæmorrhage with a renewal of cough and expectoration, at any moment that accidental causes should determine a renewal of fullness of the pulmonary vessels. Careful regulation of diet, and exercise, with mild tonics, constitute the best treatment.

